# Community- and Data-Driven Services for Multi-Policy Pedestrian Routing

**DOI:** 10.3390/s22124515

**Published:** 2022-06-15

**Authors:** Ioan Damian, Anca Daniela Ionita, Silvia Oana Anton

**Affiliations:** Automation and Industrial Informatics Department, University Politehnica of Bucharest, 313 Splaiul Independentei, 060042 Bucharest, Romania; ioandamian94@gmail.com (I.D.); silvia.anton@upb.ro (S.O.A.)

**Keywords:** smart cities, software services, route directions, performance testing

## Abstract

Pedestrian routing is important in a multitude of public spaces, especially those characterized by a large number of newcomers. Their needs may be diverse, with priority for the shortest path, the less crowded or the less polluted one, the accessibility for reduced mobility, or the sheltering from unfavorable weather conditions. Hence, typical graph-based routing must be enriched to support multiple policies, at the choice of each person. The paper proposes a systemic approach and a set of services for orientation and accessibility, which are both community-driven and data-driven, for correctly perceiving the routing necessities and the surrounding situation. The response time to a pathfinding query depends on the types of policies applied and not only on their number, because each of them contributes to the customization of the weighted graph, although it refers to the same physical space traversed by pedestrians. The paper also presents results of loading tests for up to 5000 Virtual Users, inspired from real-life requirements and executed on a graph that models a real building in our university; different policies are applied to assess performance metrics, with simulated community feedback and sensor data.

## 1. Introduction

The applications that provide drivers with real-time directions about their navigation routes are a state of practice. However, pedestrians mostly rely on traditional orientation methods, although getting to the destination in large public spaces such as transportation hubs, commercial centers, and university campuses, has become more and more complicated and it sometimes leads to delays with unpleasant effects. Various solutions to this problem are generally focused on specific requirements, separately treating problems such as pedestrian safety, optimum wayfinding, or routing of emergency personnel.

Typically, the role of humans is to consume services from various cyber-physical systems characteristic of smart buildings or of a smart city in general. Yet, recent approaches show that their role can be substantially extended to become contributors to the systems, by providing machine readable information, making or validating decisions, interacting with the system, or even assuming the role of actuators [1]. This active presence of human agents leads to the development of socio-cyber-physical systems (SCPS). Such an example is given in [1] for planning the evacuation routes in emergency situations, when relevant information should not only be acquired from sensors, but also from people in the affected area. The human inputs and their contributions to make and follow decisions may be integrated into the overall controlled system in a large variety of ways, as inputs, perturbations, actuators, or feedback. People can thus intervene in multiple ways in the coupling between perception and action [2]. 

The research presented in this paper is placed in the context of providing navigational and accessibility directions to persons inside a public space, e.g., a university or a corporate campus. This is important for offering visitors customizable experiences, by considering a range of possible needs and preferences as inputs of the routing services. It mainly targets newcomers, who require navigational information paired with customization criteria. Relevant examples are available for smart campus models that must be person-centric [3], where the benefit to people is put above other technology-driven reasons. Software services allow people to acquire a more accurate perception of the public space, and also to stay in contact with other people. This kind of perception has similarities with the case of robots that need to navigate in the same environment as people [4]; the idea is that a person’s perception and actions are influenced by other persons in the same environment and the technology embedded there. Another area that impacted our study is the intelligent decision component; a person-centric smart campus application should provide the user with the safety and understanding of their surroundings, especially in the case of navigation and access—an idea that is more broadly approached in [5].

We investigated solutions to help people with respect to their specific needs, for example the impossibility to use stairs or to go through narrow doors, the preference for a less crowded or a less polluted path, the necessity to avoid rain, etc. These concerns of accessibility and epidemiological safety are timely and need to be treated in an integrated way. Nonetheless, we want these services to be community-driven, an approach that has been widely adopted for car routing. A public space topological map contains a limited number of access points, as entrances. One can consider this as a mathematical finite set, because it is difficult to add “real” resources to infinity in a public space that is already established; for example, a new room cannot be easily added inside a building, as it would require it to be built physically. Nonetheless, the layout of a public space can withhold a multitude of configurations, i.e., a building can be renamed, a large room can be divided in order to have more laboratories, etc. It is also possible to have various preferences or limitations that further complicate the routing, e.g., someone with locomotory issues cannot follow the same path as the others, or, in case of the COVID-19 pandemic, people need to interact less and still conduct their daily duties. We want to address these diverse criteria in an integrated way, but still allow flexibility with respect to people’s choices. This is possible based on a general-purpose graph modeling that is dynamically adapted according to various considerations such as: taking into account the feedback from other pedestrians walking in the same public space; trying to avoid crowded areas at all costs; avoiding any polluted areas based on data originating from sensors; specifying that one cannot get wet in the rain and asking for real-time data from weather APIs; and not being able to use stairs because of temporary or permanent special locomotory needs. They correspond to policies that can be applied to the routing services; when a large number of pedestrians request routing directions in multiple manners (with 0-policy, 1-policy, 2-policy, etc.), the service recalculates the weights inside the graph for each invocation, so each person has a particular output tailored by his or her special needs.

Subsequently, the paper presents the related work in Section 2, and the software services for multi-policy pedestrian routing in Section 3. They are based on a graph-based model of the public space, with multicriterial weights. On top of the classical routing, we add policies that are specific to the pedestrians’ interests and are combined at their choice. The routing policies proposed to the service consumers are described in Section 3.4:

- Policy 1. Take into account the community votes.
- Policy 2. Avoid crowded areas.
- Policy 3. Avoid polluted areas.
- Policy 4. Shelter from unfavorable weather conditions.
- Policy 5. Consider accessibility for reduced mobility needs.

Several examples of algorithms for multi-policy routing are given in Section 3.5 and the evaluation method is described in Section 3.6. Section 4 presents the results of loading tests for 1000 to 5000 Virtual Users, with 0-, 1-, and 2-policies applied. Section 5.1 explains the reasons for considering these loads, considering the analysis of real-life situations, and Section 5.2 interprets the values obtained for the performance metrics evaluated. 

## 2. Related Work

By studying the scientific literature, one can identify three main research topics related to the work presented in this paper: route planning; mobility and orientation in a public space (particularly a campus); and crowd management for preventing the spread of diseases (especially COVID-19).

### 2.1. Route Planning

Route planning is generally made between remote locations that do not have the same address. It is important for our study because this application domain has accumulated a lot of experience. There is significant scientific work regarding the algorithms for navigation using GPS (Global Positioning System); most of them are based on analyzing and processing graph data for maps, but also other real-time data regarding crowds, traffic jams, accidents, and other events that may influence the selected route. Most of the time they are multi-objective algorithms, trying to find a route that will not only minimize the distance, but also the time, or a route selected to go through certain points on the map (touristic objectives or other locations). The algorithms in this area are oriented toward four types of travel: wheel vehicles (cars, trucks), railway (trains), sea travel (ships), and foot travel (pedestrians). For each of these types we selected an example characteristic to the current trends in research.

**Road vehicles**. For wheel-based vehicles it is important to have a multi-objective route planning; this may be implemented using a swarm intelligence graph-based pathfinding algorithm (SIGPA) [6], having the advantage of solving and developing personalized tourist route planning (TRP). In most cases, the route is obtained after solving a multi-objective optimization problem. 

**Railways**. A simpler problem, which is also based on graph analysis, is the problem of defining the route for railways. This can be achieved with a graph-theory-based approach, for example to solve the situations related to railway interlocking [7]. The routing for railways is less complex because the railways are less complicated from the point of view of the path, and one does not find problems such as crowding. There are also similar constraints such as speed limits, unavailable routes, and different real-time aspects (such as the occurrence of an accident). For railways, there are also specific problems such as a fixed travel time that must be achieved, or interlocks. Route interlocks are in most cases listed by signaling engineers; in [7] a graph-theory-based algorithm is used to locate all routes from a given station in such way that a new route can be obtained automatically when a station is modified. This offers the advantage of saving time and money in the process of route planning.

**Water Transportation**. In the case of water vehicles, a solution to the problems of slow planning and poor route accuracy can be the use of a route planning method based on a multi-scale visibility graph for autonomous ships [8]. This method has the advantage of using optimal routes for autonomous ships, but the disadvantage that it cannot be used for nonautonomous ships due to the poor integration of automatic commands in ships with crews.

**Pedestrian Mobility**. For the fourth type of travel, the foot travel of pedestrians, one can see that the preferred method for path planning is also graph based. In this regard, Kielar et al. proposed a unified pedestrian routing model for graph-based wayfinding built on cognitive principles [9]. The difference from the other types of travel is that for pedestrians, in order to model the realistic routing behavior, both the spatial and the social cognitive aspects must be taken into consideration.

### 2.2. Mobility in a Public Space

A large public space, such as a commercial center, an important transportation hub, or a university campus, may have a single postal address, but it requires one to move inside indoor and outdoor areas, with an architecture having a high degree of complexity. This application domain is also targeted by our work. For the mobility and orientation inside a public space (particularly a campus), there are several ways to go from one place to another: using bicycles, electric scooters, personal electric vehicles [10], or traveling as a pedestrian. The problems are usually related to pathfinding and route generation for helping freshmen (students in the first year of study) and visitors to reach a location of interest. Nonetheless, another specific issue is route generation for people with disabilities, e.g., visually impaired persons [11], or people who move using a wheelchair.

When discussing path planning in a university, corporation, or hospital campus, this includes navigating inside buildings with multiple levels and navigating between building. In order to solve this problem, one can employ a hierarchical indoor visibility-based graph (HiVG) for navigation guidance in multi-story buildings [12]. WRLD is an example of a specialized software solution for navigation and orientation (indoors and outdoors) in real world environments [13]; it uses indoor positioning systems (IPS) that provide one’s location inside a building related to where one wants to go, and what steps are needed to reach that destination. IPS can theoretically be implemented with GPS, although GPS is mainly provided by satellite signals that are easily blocked by walls. Hence, there is a need for Bluetooth Beacons, receiver antenna arrays, Wi-Fi, and short-range radio Ultra-Wideband. These devices have to be mapped to real-life structures with all their substructures (floors, rooms, etc.). The final user needs a visual tool, mostly implemented on a mobile device. Usually, sites that benefit from indoor navigation belong to a diverse spectrum, depending on the environments, such as hospitals, office buildings, shopping malls, university campuses, and airports. All of them have an underlaying topological complexity. Thus, converting a traditional infrastructure to one compatible to guided navigation involves a high cost (technical wise and material wise).

There are multiple solutions for underlying issues related to the navigation issue. An example is to use an indoor positioning system using a K nearest neighbor algorithm against a floor map layout that offers the user coordinates used as inputs in Wi-Fi data integration, with pseudo-odometry [14]. In addition, such a system could be improved by using a multi-threshold step detection algorithm. Prandi et al. presented the findings of the University of Bologna, who successfully deployed a wayfinding system that uses an Internet of Things (IoT) infrastructure in conjunction with a mobile application, providing users who belong to the university community with routing functions inside the campus [15]. In this paper, the results showed that participants were pleased with the functionality provided, which includes indoor navigation as well; the paper noted that indoor navigation is crucial, especially for people suffering from disabilities (locomotory or visually), but it can also help persons in a new, unknown place. Another approach to the same problem was based on a different technology, via Bluetooth receptors and Beacons [16]. Beacons are installed inside or on buildings to emit signals, providing geographic positioning with some imprecision in conjecture with a Bluetooth device’s range. The imprecision can be managed based on the building layout and the user’s position, to provide guidance and to store the users’ movements. The system was built with the aid of an Android OS app (“Find Me!”) that can capture a Bluetooth Low Energy (BLE) signal provided by the Beacon, then guide and locate the user through a map displayed on the user’s smartphone. This solution has the advantage of a low-cost implementation with an acceptable accuracy.

Even if not directly related to navigation or positioning, an interesting phenomenon about the perceptions and the interaction between people, and people and things, was observed for an IoT solution based on environment variables transmitted with Long Range (LoRa) technology [17]. The data were stored, processed to find underlying patterns, and visualized to develop a perception of the environment. This approach was used in the ISCTE-IUL University Campus where this 3D IoT representation was provided on mobile devices, and helped the campus reach sustainability goals. The IoT technology is used in multiple related applications, as summarized in [18], where one also proposed a SpecTalk platform to build the code necessary for IoT application against the specifications of the Taiwan Association of Information and Communication Standards (TAICS). The program built by the platform accommodates the Application Programming Interface (API) for the devices under test, which is then used by SpecTalk to test devices with TAICS data formats. IoT should be seen as a method to communicate with smart objects, but smart platforms may be needed to manage all these devices. Yet, a long-term deployment of IoT devices inside a university campus may raise certain challenges [19]. They are of technical nature, or they concern data quality issues related to data analysis in interaction with the physical space, including the idea of opening the access to the generated data for educational purposes. 

The idea of a smart university campus is approached by Fortes et al., who presented a framework for integration and representation of novel architecture, furniture design, flora, environment sensors, and communications under the name of “Smart Tree” [20]. A smart campus must be constantly monitored to assure that the functionalities are working as intended and maintenance is performed consistently, and this can be managed with various technologies [21], such as IoT and artificial intelligence. In the study presented in [21], concepts from civil and electrical engineering were enabled to develop a universal and modularized long-term sensing system. The university used for this study was the National Taipei University of Technology. A study that approaches similar issues is [22], having results obtained by a planning simulator of a campus spanned across 26,000 m^2^, using Low-Power Wide-Area Networks (LPWANs) to provide low-cost, low-power connectivity consumption. This also makes the case of the energy management issue, with solutions such as a “microgrid” campus to provide sustainable, economical, and reliable energy [23]. The microgrid would be built by distributed generators of AC, DC, hybrid loads, and an energy storage system. 

The influence of the users’ perception, this time related to air pollution, has also been approached and implemented as a mobile application and a website that support report gathering from users, as well as visualization of these reports [24]. The application uses dedicated sensors for air quality monitoring, and the data acquired from them are processed with machine learning techniques. Cloud technologies and edge computing can also play a significant role in the overall adoption and development of smart campuses, as noted in [25] concerning the problem of centralization versus decentralization in the case of data gathering from devices, underlining that performance is dependent on the system design and has a significant impact on emergency detection; the study also proposed various design options with the aid of edge computing and containers for smart building monitoring systems.

In large university campuses, a mobility plan should be defined; this can be performed for universities located in towns but also outside them. Such a plan offers suggestions to satisfy the mobility needs of students and university staff [26]. It can include different methods of transportation and recommendations for implementing a sustainability strategy to reduce car use and encourage the use of public transportation or other green transportation vehicles [27]. In order to ease the usage of a mobility plan, an electronic service can be defined and implemented as a mobile application. Such a mobility-as-a-service app [28] can present the route to follow and the transportation method and can show other information related to the facilities available on the campus, as well as the events ongoing or planned for the next period of time. This kind of approach, along with other complementary measures, allows universities to migrate to a “green” footprint.

The interface with the user is on mobile phones most of the time, but in some situations, in order to reduce the costs, a set of embedded indicators can be used for outdoor or indoor navigation [29]. On the other hand, the advantage when using the mobile phone as an interface is that other additional features can be added: augmented reality showing valuable information (for freshmen) [30], or a dialogue-based guidance system [31] that can also be good for visually impaired people that can use audio commands to direct their movements [11]. This category of handicap is an extreme case of how the system must be used by the end user. An extensive review of technologies for indoor navigation for the visually impaired is given in [32]. The discussion was conducted in terms of precision and scalability (two core concepts for the navigation of visually impaired users). There are numerous technologies available, relying on the aforementioned IPS, such as radio frequency (range-based, range-free), inertial sensors, sounds (audible sound, inaudible sound), light (visible light, non-visible light), computer vision (cameras fixed to the scene, mobile cameras), and hybrid indoor positioning systems (RSSI-IMU hybrid systems, RSSI-Vision hybrid systems, IMU-Vision hybrid systems, RSSI-IMU-Vision hybrid systems). They are supported on smartphones, which are highly available and popular devices. They have shown significant advances in terms of accuracy and speed, but the problem of positioning in indoor environments is not entirely solved; the end user needs better and less sophisticated methods to obtain a general direction towards the destination, taking into consideration a reasonable spectrum of criteria.

### 2.3. Avoiding Crowds

A more recent topic is related to disease control, and particularly the COVID-19 pandemic. It is especially relevant for public spaces, where crowds are very likely to form. This problem introduces a supplementary set of constraints regarding the path planning and tracking, whereas is does not fundamentally modify the algorithms and methods used in route planning in public spaces such as campuses. Of course, when trying to control the spread of a disease one must impose a set of restrictions to the traffic; in this case, one refers to the pedestrian mobility and not to the transportation vehicles.

University campuses are places where COVID-19 transmission can have a high impact on society due to the mobility of the affected persons. A set of studies have been conducted to see how the transmission is carried out and to determine the right measures for prevention and distancing. They regard the dependency between pedestrian dynamics and epidemiology [33], the aerosol transmission [34], the mathematical modeling of disease spreading [35], and the risks of students’ exposure to COVID-19 in a university building [36] or in other urban settlements [37]. 

From the point of view of mobility and route planning, disease control influences the way such algorithms are defining the routes, e.g., by indicating different routes for pedestrians inside buildings (an entry route and a different exit route) or by implementing pedestrian distancing and crowd control. For example, Durán-Polanco and Siller proposed a recommendation system using a behavioral game theory and agent-based models [38]. Geneletti et al. simulated policy scenarios for different restrictions, to assess potential crowding of green areas [39].

## 3. Services for Multi-Policy Pedestrian Routing 

Our research is focused on services that assist pedestrians finding their way through a public space, let them choose the criteria they are most interested in, and advise them on the most appropriate route given their needs and the flow of people from that moment. Section 3.1 presents the service-oriented design at a high level of abstraction; Section 3.2 describes the algorithm for finding pedestrians’ routes, using on graph whose weights are computed based on multiple criteria. Section 3.3 explains the data structure for modeling a public space as a graph that may be used by the routing service, and Section 3.4 shows how the graph weights may be computed given a set of policies from which the pedestrian who uses these services can choose. 

### 3.1. Service-Oriented Design

**Figure 1:**
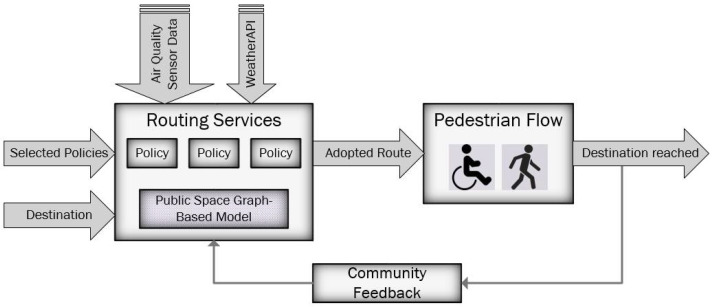
Pedestrian routing system.

Our design is based on exposing services for wayfinding, to help people choose the most appropriate route for walking through a public space and getting to their desired destination. The core of the system is the capability to model the real-world space in a graph-like structure, to enable the adaptability of routing algorithms, as detailed by Costa et al. in [40]. The route adopted by each person has an influence on the overall pedestrian flow, and the routing services also take into account the feedback given by the community of people who benefit from such services (Figure 1).

The system supports different kinds of users, such as administrators, cleaning personnel, employees, guests, emergency personnel, etc. The participant is authenticated and authorized by A&A (authentication and authorization) services before using other exposed services. The role-based approach was inspired by Linux file permissions and access [41]; it is important since the state of the whole graph is secured behind a series of enforcements that holds the system in a robust state, being a part of the identity and access management strategy [42]. The permissions to create and maintain the graph-based model are only given to administrators who maintain the public space model via specific services. The non-administrative users can use the routing service to visualize the graph nodes representing various locations in the public space, generated by the administrators; this is a management aspect as well as a security aspect [43]. Specialized users, such as cleaning personnel, can use soft maintenance services, which do not change the structure of nodes but allow one to change the sanitation status, which is especially needed if one considers the protection against hazards such as pandemics.

The routing service takes as input the nodes generated by administrators and external parameters, such as weather, epidemiologic risk, personal issues such as locomotory difficulties, or emergency situations. For this reason, the nodes must be created in conformity with a model that supports such policies; therefore, these policies need to be known before the actual real-world resources are modeled as nodes. For the system to be more robust, these policies represent a resource inside the system, exposed by other services; they should be a source of compliance for the nodes that are created by invoking them. The services are written to expose HTTP endpoints so they are to be invoked in a client–server architecture with mobility in mind; navigation should be available on mobile devices, since this service consumption is ad hoc and instant. The routing service also calls other specialized services for route finding, edge management, vertex management, graph management, and graph entrance management. Supplementary details about the model and the routing algorithms are presented in the following sections.

### 3.2. Route Finding Based on Multicriterial Weighted Graphs

A typical manner in which to represent a real-world public space is a graph, since graph theory already provides algorithms that follow the behavior in the real world, where navigation from the current location (node) towards a destination (node) is a set of steps that can be modeled with graph vertexes. 

A one-criterion routing algorithm operates based on a weighted graph that only takes into consideration the amount of consumption that it takes to traverse the resources (nodes). Let us analyze an example of a weighted graph, where A, B, and C are nodes and *X*, *Y*, and *Z* costs to navigate from A to B, B to C, and A to C, respectively. If, on the route A → B → C, the cost is *X* + *Y*, and it is more economical than choosing A → C that costs *Z*, then the chosen route should be A → B → C.

**Figure 2:**
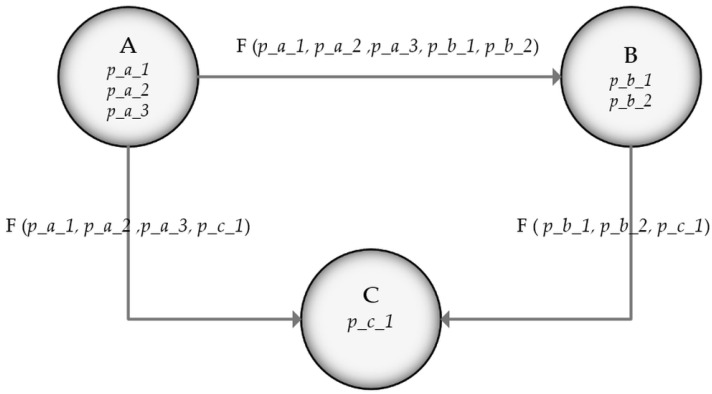
General weighted graph for multicriterial routing algorithm.

This approach can be employed as a multicriterial algorithm. The changes towards the multicriterial algorithm must be reflected both on the algorithm and on the representation; the nodes should have special properties that the algorithm should take into consideration. Thus, the weights of the dependencies (vertexes) become functions of the properties of the connected nodes, as represented in Figure 2. What makes this approach a multicriterial one is that some properties of the nodes might not be interesting for certain stakeholders, and the algorithm should treat those properties as “null”. The nodes must have multiple properties that represent the interests of certain users; such as for a newsletter subscription, all people have interests, but often they are different from one to another, and should not impact other people’s interests. A user may “call” the multicriterial algorithm with his or her particular interests (nodes properties); thus, the multicriterial algorithm calculates the output in a customizable manner.

In a general weighted graph with three nodes, each connection between the nodes has a cost known as weight in the graph theory. In the multicriterial approach, one can assume that there is a function cost for computing the weight. Figure 2 depicts a weighted graph with three nodes, in a similar connection configuration as in the example above. The difference is that the cost to traverse nodes is no longer standalone information. Each node has a few properties, e.g., A has three properties: *p_a_1*, *p_a_2*, and *p_a_3*, etc. These properties on each node represent how difficult it is to access this node. To traverse from one node to another, a cost function that integrates the properties of the two nodes must be taken into consideration, i.e., to go from node A to node C, one must pay the cost F(*p_a_1*, *p_a_2*, *p_a_3*, *p_c_1*) since A has the cost properties *p_a_1*, *p_a_2*, *p_a_3* and C has the cost property *p_c_1*. This approach lets us represent how hard it is to access a resource and to abstract the cost between two such resources. We consider that each of these properties has a numerical value, and the function F is the SUM function, meaning that it adds all the properties and virtually changes the weights of the graph. If these properties change dynamically, the weights of the graph also change dynamically. If we abstract these properties under certain categories (or criteria), the users can call the multicriterial routing algorithm giving these inputs. This means that the user wanting to navigate in the building/campus/public space can minimize the impact for the given criteria (since the base algorithm minimizes the navigation cost). The multicriterial algorithm retrieves the value of the properties from the nodes that have the desired properties and, for the nodes that do not have them, the algorithm treats them as if they do not add anything to the cost (the correspondent value is 0).

### 3.3. Graph-Based Model of the Public Space

Let us consider that there are multiple buildings inside a public space, thus multiple jurisdictions, usually managed by the buildings’ administrators who can provide the information for transforming the real-world objects (entries, hallways, rooms, etc.) into a general-purpose graph for which one can employ a multicriterial navigation algorithm. This bootstrap phase allows administrators to create nodes with properties and connections between them. A node is amorphous, meaning that it can represent any object, as indicated above (entry, hallway, restroom, etc.). The administrator is responsible for the granularity of the representation (a hallway can be represented as one node or multiple nodes, depending on physical limitations or local considerations). The connections (vertexes) are links between nodes that are important for the multicriterial routing algorithm and should also be managed by administrators. Connections can be indoor (connecting nodes inside buildings) or outdoor (connecting nodes outside of buildings, usually between different building’s entrances). 

**Figure 3:**
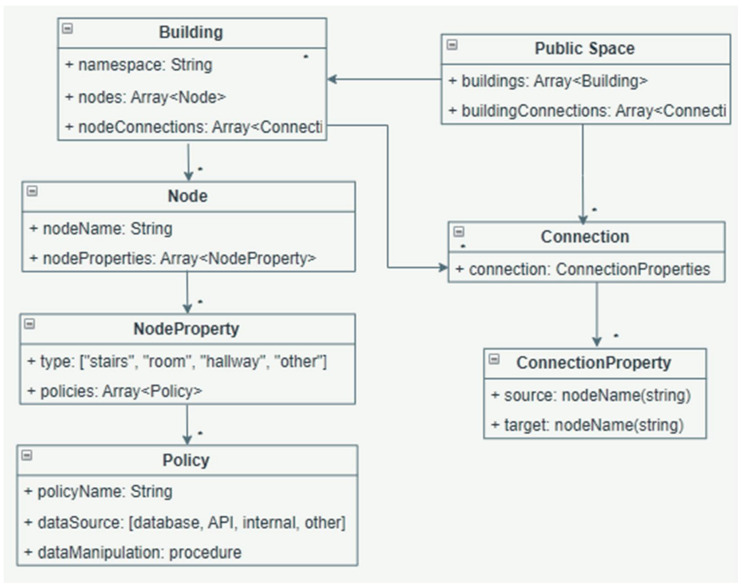
Building graph model with policies attached.

The building graph model, represented in Unified Modeling Language, is given in Figure 3. A public space includes a number of buildings and a number of connections between the buildings that are on-site. A building’s attributes include namespace (i.e., the name of the building), the number of nodes (correspondent to architectural features), and the number of connections between the nodes in the same building. A connection has two properties (source and target) to represent the neighbors of a node or building. Each node has node properties (see “*” to show the multiplicity > 0 in Figure 3), including the type of the node (hallway, stairs, room, etc.) and policies. A policy is defined by name, data source (where the data come from, to build the particularities of the policy), and “dataManipulation” (a procedure for how to interact with the data from the data source).

As explained previously, a node’s properties should be grouped under common criteria, to provide the user with the possibility to invoke these criteria when calling the routing algorithm (otherwise the algorithm cannot centralize information and create the weights on the graph); thus, one should employ a specific abstraction to describe this requirement. For this purpose, we define a series of policies to be available for the pedestrian who wants to obtain routing directions to reach the desired destination. A policy, according to the Cambridge Dictionary, is “a set of ideas or a plan of what to do in particular situations that has been agreed to officially by a group of people, a business organization, a government, or a political party”. The policies are defined in our approach as rules on how the multicriterial algorithm should compute the weights on a graph, where to retrieve or store information about nodes, and how information from nodes should be used while the multicriterial algorithm is running. 

Such policies are applied to the entire graph and then they activate certain properties for each node; the attribute “nodeProperties” from Figure 3 can be populated with data with respect to the chosen policies, to be then considered in the cost of each connection, by using the SUM function on all data quantified from policy data. In this way, the algorithm minimizes the overall cost and outputs the least cost route. Nonetheless, other properties of nodes can be populated with access data that will be provided to the user as the route generator output.

### 3.4. Routing Policies

Typically, a route generating algorithm for a graph follows the principle of reducing the cost of travel; this can be realized as the shortest path, i.e., traversing as few nodes as possible, or as the most cost-efficient path, choosing the route with the least cost to arrive at a destination. Our algorithm adopts the cost-efficient approach for the following reasons:

- A cost-efficient path algorithm works on top of a weighted graph.
- A weighted graph can be created to quantify the difficulty of accessing a node.
- Since we want to build on top of a weighted algorithm, we came with solutions to integrate more diverse information (based on the users’ needs) in the weight value for every vertex; this diverse information has to be used in a predictable manner by the navigational service, and we categorize it in terms of policies. The policies may be optionally used as inputs in the call of the routing services, to determine how to update the weights in the graph, and then process it based on a cost-efficient path generation algorithm.

We decided to look at a node as a stand-alone concept such as a web page, and let the node dictate how hard it is to be accessed, based on policies that are attached to it. The path generating algorithm looks at every node in the graph and decides, based on the policies given at the algorithm call, if passing through that node is desirable or not. These policies are quantified and provided as a weight addition in every vertex that the node has, allowing us to use well-established graph path generation algorithms. Policies can be used as methods to integrate data taken from multiple sources, other than the original graph model of the public space, which is essentially static. Policies are the dynamic aspect of the platform and of our data-driven routing system because they enable the end user to check which are the most important aspects to be taken into consideration for a personalized navigation. Data inputs that are considered to control the pedestrian flow via policies can originate from people, sensors, and external providers. Below we describe the policies identified as mandatory to fulfill the users’ needs, but other policies (with other scopes) can be attached to the same nodes. 

**Policy 1. Take into account the community votes.** A voting policy can offer to the general user the ability to vote if a resource respects certain criteria. For example, one can vote if an entrance in a building is open enough for people with wheelchairs. Any user can vote how this entrance respects this criterion; the votes in time add up and are quantified via a mean value. This mean value can be used to increase the weight value of any vertex that this entrance has. Who decides to take into consideration the voting mean value? The answer is the user, because the user specifies (through the inputs of the routing algorithm) if this policy should be considered. If the user does not choose this policy, it is considered of no interest to him/her. 

**Policy 2. Avoid crowded areas.** Another policy, also driven by the community of pedestrians using the routing service, takes into account the crowding factor. When users ask the system to obtain a certain path, it means that, when accessing the constituent real-world locations, they occupy the correspondent graph nodes. Many users might need to traverse some sections in the real world, which are overlapped with other users’ sections, resulting from their needs to find their way; therefore, crowds can form spontaneously. This case is resolved by allowing users to use a crowding-factor policy, which provides them with the least crowded routing path. The implementation for this policy is based on the outputs of previous users’ routing needs, by increasing the weight on the constituent nodes. Thus, when the next user calls the routing service with the policy to avoid crowds, the weights on the links of the previously visited resources are increased, and the algorithm provides an alternative route that is more cost-efficient (where the cost regards how crowded the space is). After a given time interval, the weights on the nodes are automatically decreased if no longer provided by the routing service, meaning that the correspondent locations become less crowded.

**Policy 3. Avoid polluted areas.** This policy is data-driven and can be applied if the public space is provided with sensing devices to detect various physical quantities to characterize the air quality. For this purpose, one collects data from sensors installed in various locations of the public space; a communication channel between our services and the sensing devices must be established. When a user chooses the policy for polluted areas, the routing service obtains data from the sensing devices and quantifies if it is necessary to increase the graph weights accordingly.

**Policy 4. Shelter from unfavorable weather conditions.** This policy also ensures a data-driven routing, based on data originating from external providers, such as weather forecast platforms. These external providers can be called from our service on a specific communication channel when this policy is invoked; hence, the routing service takes into consideration minimizing the effect of external factors, for example to minimize the outdoor routing under hot weather received from a weather forecast, or under heavy rain. Currently, the weather states considered are sunny, cloudy, rainy, snowy, windy, and blizzards.

**Policy 5. Consider accessibility for reduced mobility needs.** This policy holds information about the accessibility of the public space locations that may create difficulties to users with reduced mobility needs. When this policy is used in the routing algorithm, the calculated route eliminates nodes or connections that lack accessibility, or at least the user is presented with information regarding the potential risks in the real world (stairs with many steps or without railings, narrow doors, lack of ramps, etc.). This is also performed by increasing the weights in the correspondent graph.

### 3.5. Multi-Policy Routing Algorithm

The multi-policy routing algorithm uses a computational object modeled as a weighted graph that represents the topology of a real-world campus, or public space in general. The cost-efficient routing algorithm is based on the graph’s weights. To achieve multicriterial navigation in this graph we enhance the properties of each node, with correspondence in the real world. Each property evolves based on the policies selected by the end user, because each user has different needs and preferences, which are expressed by selecting a set of policies. Each user, when requesting the routing services, indirectly invokes a graph loaded with the user’s particularities; the graph pathfinding is community-driven and data-driven, with respect to the selected policies. Consequentially, the output route is particular to the individual user.

**0-Policy Routing Algorithm.** By default, the underlying graph of a building has the weights of all connections equal, because the nodes represent physical locations that are very close to each other. This applies in the case of a 0-policy routing request, where the cost-efficient algorithm prioritizes the path that cumulates a minimum value, i.e., the shortest path. Nonetheless, the users indirectly increase the weights on the paths that were given as routing solutions if the user follows the routing directions. This applies to 0-policy routing as well, because in case a user wants to avoid crowded places, it is necessary to know what locations (graph nodes) were the most visited, even if the users who requested the routing service have not selected Policy 2. If a node is present in a routing path output calculated in the recent past, the crowding-factor property is increased with a fixed amount; if a node has not been present in an output routing path, the crowding factor is decreased with a fixed amount. 

**Multi-Policy Routing Algorithm.** In the case of the multi-policy routing algorithm, each node has specific properties that follow the policies, and these properties are used to update the weights between nodes. The cost-efficient routing algorithm outputs the path that cumulates a minimum value, which may not always be the shortest path, as it was in the case of the 0-policy request, but the one that is the most convenient to the user given his or her needs. Each policy looks at the specific node property/properties that indicates it. The policy looks at the paired properties on each node; Algorithm 1 shows that the system always listens to the user’s requests and delivers the output of the routing service calls. Certain details are necessary to compute the nodes’ properties:

- *decreaseCrowdFactor*—how much a *crowdProperty* on a node should decrease after the *crowdTimeframe* expires.
- *crowdTimeFrame*—a time interval after which one considers that the *crowdProperty* value on a node should be decreased.
- *increaseCrowd*—the amount that the *crowdProperty* value on a node should increase after it was given as a routing solution and the directions are followed by the user.

**Algorithm 1.** Multi-policy pedestrian routing algorithm**Inputs**: *increaseCrowd***while** routing service provided **do****get** from user call: *start*, *finish*, *policies***for***policy* in *policies*:**switch***policy*:Policy1: **call** community feedback algorithmPolicy2: **call** crowd avoiding algorithmPolicy3: **call** pollution avoiding algorithmPolicy4: **call** weather conditions algorithmPolicy5: **call** reduced mobility algorithm**end switch****end for****call** multicriterial route finding algorithm**send** directions to user**get** from user call: *accept directions***if***accept directions* **for***element* in *path*
**do***crowdProperty* = *crowdProperty* + *increaseCrowd**crowdTimestamp* = *currentDate***end for****end if****end while**

When the user calls the routing service, the system takes from the call details for *start* (the point from which the navigation begins) and *finish* (the destination of the navigation). One computes the *path* against the newly updated graph taking as input *start* and *finish* and sends the user the result obtained with the multicriterial routing algorithm. After this, for each node in the *path* given to the user, the *crowdProperty* for this node is increased with *increaseCrowd* (configured to tell the *crowdProperty* by how much it should be increase after it was visited), and one updates the *crowdTimestamp* of this node to be equal to the *currentDate*.

Further on, to exemplify the multi-policy routing algorithm, let us analyze the case of a 2-policy routing, applying Policy 2 and Policy 3.

**Crowd Avoiding Algorithm (Policy 2).** As resulted from the previous description of the algorithm, even if there is no policy selected, the underlying graph is always updated with the increased weights on the sections that were most visited by other pedestrians in a certain period. This also means that, if the path with Policy 2 has been given to a user in the recent past, the algorithm should not favor this path to the next user who requires the routing service with the same policy. Algorithm 2 describes what is performed if a user invokes the policy to avoid crowded areas; for each node of the graph, it retrieves:

- *CrowdProperty*—the crowd value on the *node* (the bigger the value the most visited is the node).
- *CrowdTimestamp*—when the *crowdProperty* was last updated.

**Algorithm 2.** Crowd avoiding**Inputs**: *decreaseCrowdFactor*, *CrowdTimeFrame*, *increaseCrowd*, *graph*, *limit1*, *limit2*, *limit3, limit4, limit5, limit6***for***node* in *graph*
**do****get** from Policy2: *crowdProperty*, *crowdTimestamp**timeDifference* = *currentDate* − *crowdTimestamp**timeDifferenceFrame* = *timeDifference*/*crowdTimeFrame**decreaseCrowdProperty* = *decreaseCrowdFactor* × *timeDifferenceFrame**crowdProperty* = *crowdProperty* − *decreaseCrowdProperty***if***crowdProperty* < 0 *crowdProperty* = 0**end if****for***connection* in *nodeConnections*
**do**:**if***crowdProperty* > *limit1*
**and**
*crowdProperty* < *limit2* *addingUpNorm* = 1**end if****if***crowdProperty* > *limit2*
**and**
*crowdProperty* < *limit3**addingUpNorm* = 2**end if****if***crowdProperty* > *limit3*
**and**
*crowdProperty* < *limit4**addingUpNorm* = 3**end if****if***crowdProperty* > *limit4*
**and**
*crowdProperty* < *limit5**addingUpNorm* = 4**end if****if***crowdProperty* > *limit6**addingUpNorm* = 5**end if***weightConnection* = *weightConnection* + *addingUpNorm***end for****end for****return** graph

The algorithm proceeds with computing *crowdProperty* as the current *crowdProperty* minus *decreaseCrowdFactor*, multiplied by the result of *currentDate* minus *crowdTimestamp*, and divided by *crowdTimeframe*. In this manner, the *crowdProperty* is always updated based on how much time has passed since the node was visited. If after this operation *crowdProperty* is less than 0, it means that a long time has passed since the *node* was visited, hence it is a viable *node* to be visited for users who specifically chose to avoid crowds. After *crowdProperty* for this *node* is computed, one updates the weight for each connection of this node. The five limits may be set to adjust the weights by adding up a norming number, with respect to the degree of crowdedness considered appropriate.

As an example, let us consider *crowdTimeFrame* = 120 s (2 min) and *decreaseCrowdFactor* = 1. Supposing that a *node* with *crowdProperty* = 1 was last visited 60 s ago (1 min). This means that the new *crowdProperty* is equal to 0.5. If the same *node* was last visited 120 s (2 min) ago, the new *crowdProperty* is equal to 0, i.e., the algorithm recognizes that the location correspondent to that node is not crowded (enough time has passed since the last person traversed this node) and it is favorable to computing the new path across the public space.

**Pollution Avoiding Algorithm (Policy 3).** The pollution avoiding policy increases the paired property of the nodes based on data gathered from sensors; thus, a sensor value is mirrored on the paired node property. In the same way, as explained in the above paragraph, the weights of the node with sensor policy are updated accordingly when Policy 3 is invoked by the user, to favor the paths that contain nodes with the smallest pollution values. In Algorithm 3, called when the user selects Policy 3, one looks at each *node* and retrieves the *sensorDataSource*, which is the data source providing the readings from the air quality sensors. The algorithm calls the sensor data source, reads the *sensorValue*, and stores it for itself. After *sensorValue* for this *node* is computed, one updates each connection weight for this node. The five limits may be adjusted according to the thresholds considered relevant when measuring the air pollution; they are used for computing the *addingUpNorm*, to be added to the existing weights.
**Algorithm 3.** Pollution avoiding **Inputs**: *graph, limit1, limit2, limit3, limit4, limit5, limit6***for***node* in *graph*
**do****get** from SensorPolicy: *sensorDataSource*, *sensorValue**sensorValue* = call *sensorDataSource***for***connection* in *nodeConnections*
**do**:**if***sensorValue* > *limit1*
**and**
*sensorValue* < *limit2**addingUpNorm* = 1**end if****if***sensorValue* > *limit2*
**and**
*sensorValue* < *limit3**addingUpNorm* = 2**end if****if***sensorValue* > *limit3*
**and**
*sensorValue* < *limit4**addingUpNorm* = 3**end if****if***sensorValue* > *limit4*
**and**
*sensorValue* < *limit5**addingUpNorm* = 4**end if****if***sensorValue* > *limit5*
**and**
*sensorValue* < *limit6**addingUpNorm* = 5**end if***weightConnection* = *weightConnection* + *addingUpNorm***end for****end for****return** graph

Generally, a multi-policy algorithm needs norming. There is a weighted graph underlaying and each policy selected by the user increases the weights in the graph in a customizable manner, so one needs to decide how much each policy influences the weights. We applied an egalitarian influence for each policy, meaning that it can only have a limited influence on the weights of the graph. In theory, a connection weight can be set no matter how high, but this approach does not behave well in practice. If all connections’ weights follow a predefined limit, the preferential routing can reach its purpose. The high-level interaction with a policy is as follows: the algorithm takes data from a source that is special to the policy, those data are used as input to increase the weights of the graph, then the routing algorithm uses the new weights. A norming method is necessary in the second step, when one takes the data and uses them as inputs for the weights. Our solution proposes that each data value is mapped to a discrete value from the set {1, 2, 3, 4, 5}. In Algorithms 2 and 3, these norming numbers are added to the weights of the graph, thus representing desirability levels (1—very desirable, 2—desirable, 3—neutral, 4—undesirable, 5—very undesirable). 

To explain how the weights in the graph are updated by the user choices, in a data-driven manner, let us discuss another example. The underlying basis graph has a standard unified weight; the weight between each two nodes is *W*. For this example, let us assume that *W* = 1. One considers a model of public space with three buildings, named Building_A, Building_B, and Building_C. These three buildings have one entrance each. The layout of the public space is characterized as follows. Building_A and Building_B are connected by an indoor hallway, and Building_B is connected to Building_C by another indoor hallway; Building_A is not connected directly to Building_C, but their entrances are close to each other. If a user would like to go from the entrance of Building_A to the entrance of Building_C, the path returned by the algorithm is the shortest one: “entry of Building_A → outside → entry of Building_C”. If a user calls the navigation algorithm with the weather policy attached for the outside part of the public space, and if the weather is less favorable to traversing outside, then the weights of the edges connected to the outside locations are increased. The weather policy queries a weather API that provides the status, which is mapped to a member of the set {sunny, cloudy, rainy, snowy, windy, blizzard}. Then, the policy must also implement the mapping of these values to the interval 1–5 (where 1 represents favorable weather so the outside path can be used, and 5 represents unfavorable weather); as the blizzard is mapped to 5, if a user calls the routing algorithm with the weather policy, then the weights in the graph are recalculated to mirror the real situation, so the weight of the outside edges is *W* + 5 = 6 (in this graph there are two edges with the outside). The routing algorithm provides the user with the safest route for him/her: “entrance of Building_A → entrance of Building_B → entrance of Building_C”, which in total has a cost of three, compared to the alternative route: “entry of Building_A → outdoors → entry of Building_C”, which in this case has a total cost of 12.

### 3.6. Evaluation Method

The development of these routing services relies on the Service-Oriented Architecture (SOA) and the webservices are based on a RESTful approach. The administrators can access an interface to map the real-world resource objects (that represent points of interest, i.e., physical locations that are important for the public space) to the computational ones inside the graph. We refer to these computational objects as resources, which are dynamic objects that can be interrogated and updated, but they represent a real-world object at the same time. It is first important to establish a general-purpose lightweight graph model, in order to have the option to easily adapt the resources in the future. This general-purpose graph operates with concepts such as resources and dependencies (nodes and vertexes, respectively). The resources need to have the possibility to evolve in time and provide information to the stakeholders. The configuration provides resources that support multiple properties, based on all stakeholders’ needs (policies). We shifted from a standalone cost (which in the end has to be a particular value stored in a MongoDB database) to a cost integrated in the resource properties to store it with other properties a physical resource might have in a database, and to expose it as a resource on REST webservices. The connection data remain unaffected.

For the scope of this evaluation, Policy 3. Avoid polluted areas, contains a mocked service that, when called, retrieves data later used as input for the calculation of the new weights in the graph. This mocked service provides a random number between 4 and 20 (inspired by the typical current output of an air sensor 4 ÷ 20 mA); this number is treated as a real input and does not change the logic of the multi-policy algorithm. In a real-life scenario, the mocked service that retrieves data can be changed with a service request to a collection of sensor data, or to the application of a webservice-based sensor. The purpose of Policy 3 is to point to the correct data source and to provide the logic on how it should be consumed; a discussion on the five limits that may be chosen is given in Section 5.2.

For the testing scope, we modeled a building in our faculty with the method described in the sections above. The resulted graph contains 107 nodes and 338 connections between them. The performance tests were executed for 1000, 2000, 3000, 4000, and 5000 Virtual Users (VUs); the selection of these numbers of users are further discussed in Section 5.2. These VUs are spawned in the system with the total number desired for VUs, divided by 10 per second. For example, when testing the system with 1000 VUs, the spawn factor is 100 VUs/s, for 2000 VUs it is 200 VUs/s, and so on. After reaching the desired number of VUs in the system, the test continues for 60 s, and after that the VUs are evicted from the system; thus, the system does not receive requests any longer. The requests are sent to the system after a random time, between 0.5 s and 2 s. 

The tests were run on a machine with Intel(R) Core (TM) i5-1035G1 CPU and 8 GB RAM, to evaluate whether it can deal with the stress from real users on a real-world architectural topology, thus offering a reliable service. The users call the services to compute the route between two points, with a random start and finish taken from the nodes list. This paper presents the results for three of the test cases, executed with the setup described above:

- The 0-policy routing.
- The 1-policy routing for Policy 2. Avoid crowded areas.
- The 2-policy routing for Policy 2. Avoid crowded areas + Policy 3. Avoid polluted areas.

Software performance testing is the evaluation of how a system can be expected to perform in real life. To decide what are the best indicators for our routing service, we generally considered the category of web-based applications; for them, one typically evaluates resource usage, throughput, stimulus-response time, and queue lengths [44]. The tests defined for the three cases mentioned above measured the performance through the following metrics:

- Median response time—the most likely response time of the system.
- Average response time—how long a Virtual User waits for a response after it called the routing service; this is an indicator of the experience that the real users would have in real-life situations.
- Number of requests—how many requests were performed in the period when the system has been loaded with the Virtual Users’ calls; this indicates how many requests would be supported in production.
- Number of requests per second—how many requests the services can support.
- Failure rate—how many requests were refused by the SOA application; this indicates the experience users would have, a failure meaning that a service request does not deliver an actual path between source and destination, but an error message.

The tests were performed with Locust 1.5.3, a Python-based performance testing tool, where the tester can define user behavior through coding and employing multiple Virtual Users to execute this behavior simultaneously. The simulations were conducted in the same conditions, with both hardware and software. The model of the public space (i.e., the graph representation) was the same for all tests; the weights in the graph were dynamically driven by the user options, which translates to specific requests to the routing services.

## 4. Results

**Figure 4:**
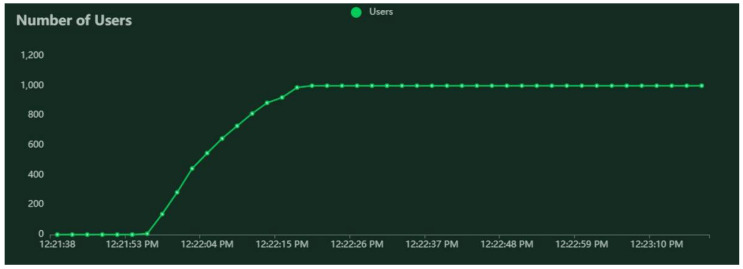
Results for 1000 VU spawn in the system and held 60 s.

**Figure 5:**
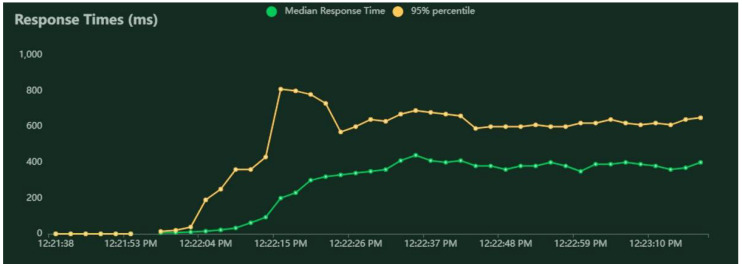
Response time for 1000 VUs and 0-policy.

**Figure 6:**
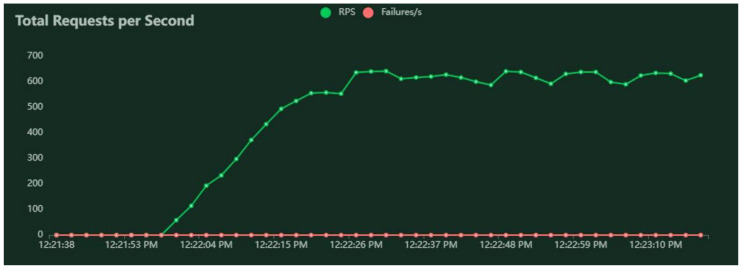
Requests per second and failure per second for 1000 VUs and 0-policy.

A series of performance tests were executed according to the method described in Section 3.6. The RESTful services for pedestrian routing were deployed on a machine with average computational performance, to assess to what extent it can deal with the load originating from a number of users and a graph topology similar to real-world situations. The results obtained for three test cases are given in Table 1 (for 0-policy routing), Table 2 (for 1-policy routing), and Table 3 (for 3-policy routing). Examples of results for VU spawn rate and holding time are represented in Figure 4, for median response time and 95 percentiles in Figure 5, and for average requests per second in Figure 6. Figure 4, Figure 5 and Figure 6 are realized with Locust and correspond to the simulation of 1000 VUs and the 0-policy routing.

**Figure 7:**
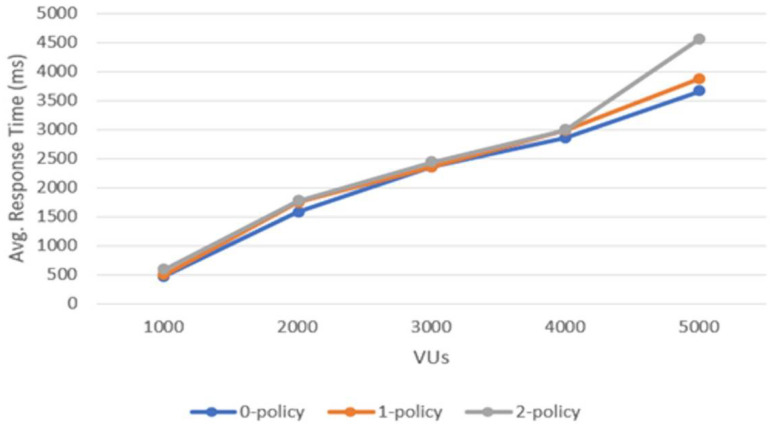
Average response time versus the number of VUs for the three test cases.

To better visualize how the services perform under different policies, Figure 7 depicts a graph that shows the average response time against VUs for the three test cases mentioned in Section 3.6. One can notice that the best average response time curve belongs to the 0-policy test; for this one, the services use the least computational effort. For the cases with 1-policy and 2-policy, the average response time curves become higher with the number of users (e.g., for 4000 VUs and 5000 VUs); however, even for this load, the differences between 0-policy, 1-policy, and 2-policy are under 1500 ms, keeping the performance still acceptable for the end user. The average response time curves are substantially close for smaller loads (for example 1000 VUs, 2000 VUs, and 3000 VUs); in many points they overlap, suggesting that for smaller loads the routing services can handle and provide the response with similar performance.

**Figure 8:**
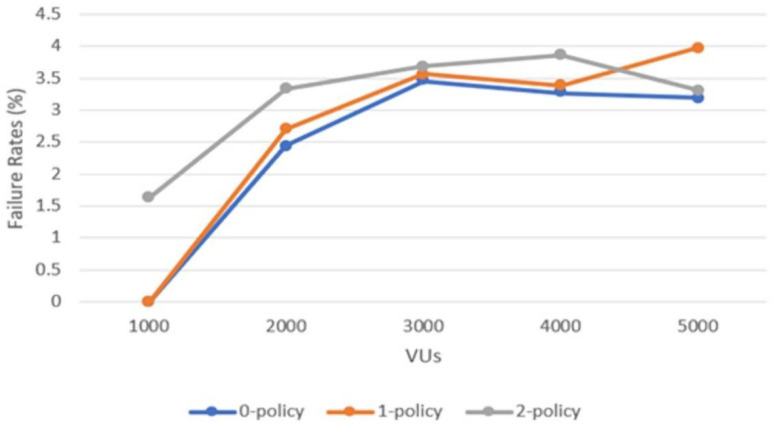
Failure rate versus the number of VUs for the three test cases.

Another factor that directly impacts the end user is the failure rate of the routing services. In Figure 8, one can observe failure rates (%) against the number of VUs calling the routing services for the three test cases considered. The failure rates are in general under 4%, and higher for more VUs. For different numbers of policies applied, the values of the failure rate are rather close to each other, proving that the system is built to handle all cases in the same manner, without a strong impact from VUs and the computational effort involved.

**Figure 9:**
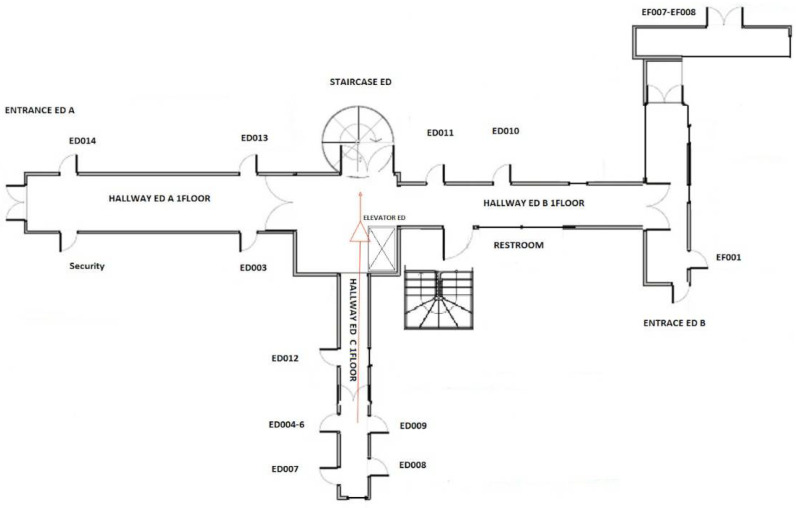
Example of graphical routing directions provided by the user interface.

As an example of results obtained with Policy 2, Table 4 shows three possible routes that were recommended after sending 100 requests in a short period, from users who prefer to avoid crowded areas. Consequently, the *crowdProperty* of each node was increased at each request, and because one has not waited a long time between requests, the *crowdProperty* of each node has not decreased (according to Algorithm 2). The test was for finding the route from a room situated on the ground floor (“ed009”) to one on the first floor of the same building (“ed114”). Between the floors one can go by elevator or up the stairs; two staircases are available. Figure 9 also shows the interface that is provided to the user for a response correspondent to Routing Variant 1, where the graphical directions represented as arrows are generated, overlaying the building floor model at a scale representation. The directions are also given in the interface as a sequence of steps, including information about the risks related to various locations. 

## 5. Discussion

Section 4 presented a series of results with regard to the routing algorithm performance, obtained with the method described in Section 3.6. This section discusses how these results may be interpreted and compared to the related work (Section 5.1), and what loads are plausible compared to real-life requirements (Section 5.2).

### 5.1. Results Interpretation 

Let us first discuss what is the meaning of the results given in the three tables from Section 4, corresponding to different routing service calls. Table 1 represents the case where all the users call the 0-policy routing service, meaning that they are only interested in the shortest path between a random start and finish locations. Table 2 represents the case where all the users call the 1-policy routing service with Policy 2, meaning that they select the option to avoid the most crowded areas; this includes decisions not considered previously in the 0-policy algorithm. Table 3 represents the case where all the users call the 2-policy routing service, wishing to avoid the crowded and the polluted areas; the data originating from sensors are simulated, considering that measurements with lower values indicate a better air quality. 

To analyze if the obtained results indicate a good user experience of the routing services, let us compare the three tables. We notice that there are differences between 0-policy, 1-policy, and 2-policy testing results but, in each case, the difference between 2000, 3000, 4000, and 5000 VUs is negligible regarding the response time; the failure rate is 0% for 1000 VUs, in the 0-policy and 1-policy scenarios, and under 4% for a larger number of users in all scenarios. The requests per second are substantially similar for all the three test cases, assessing that the routing service can handle these sorts of loads reliably. The response time indicator can be assessed in comparison with a typical web page surfing, where 1 to 2 s to load the content represent the user preference, and maximum 5 s to load the page is also considered acceptable, according to [45]. Our services are more computing extensive than a simple web page retrieval, but they remain within the same preferred values. A more modular software architecture would offer the possibility to scale different modules. Scaling and distribution can offer better performance results, especially in production [46]; moreover, the way to store the underlying graph impacts how one retrieves data from it.

With regard to the routing algorithm, there are steps that are executed to prepare the graph for applying supplementary policies; this is performed even in the absence of any policy selected by the user. Thus, no matter the manner in which the users call the routing service, the underlying graph is updated to reflect which nodes were visited; one always saves the feedback from the pedestrian community to know if a location is visited by more people and it becomes more crowded, and this information is then ready to trigger the route-finding decisions when Policy 2 is invoked. Nonetheless, we notice that each user call is unique; it takes into consideration the user’s needs by offering him or her the possibility to apply 0 or several policies. Then, in the background, each user is given a different graph configuration (the weights of the graph are different from user to user), and the routing algorithm has different computations to make; therefore, there is a high probability that the service outputs a different route from user to user. Yet, for very close locations this is not possible because topological resources are tightly coupled; for example, the path from a classroom to the hallway placed in front of it is always the same, i.e., classroom–hallway, because there is no other route to be taken.

The obtained results also depend on the way one applied the weights for the multi-policy routing and on the choice of the five limits from Algorithms 2 and 3. For example, for Policy 2. Avoid crowded areas—*crowdProperty* could increase by 1 for each accepted route from a user; for *crowdProperty* between 1 and 15, the norming number was mapped to 1, between 15 and 25, it was mapped to 2, and so on. For Policy 3. Avoid polluted areas, when a read operation is performed to obtain the value from a sensor, we map the value against predefined configurations in order to obtain a discrete norming number among {1, 2, 3, 4, 5}. If one considers a typical air sensor that has an output of 4 ÷ 20 mA, if the output read from the sensor is between 4 and 8, the norming number was considered 1, and if it is between 8 and 12, it was mapped to 2, and so on. This mapping is a property belonging to the policy; thus, a policy does not only contain information on where it obtains the data from, but on how it maps it as well. In this case, if all five policies are invoked by the user, they all add to the general weights, and the maximum weight of a connection can be 25. This maximum value of the weight does not impact the user experience with the graph, because the graph changes its inner weights dynamically; thus, a constant maximum level across all connections is highly unlikely for long periods of time.

Given the results from Table 4 (correspondent to 100 consecutive executions of the routing algorithm with Policy 2 Avoid crowded areas), three routing variants are observed. Between Routing Variant 1 and Routing Variant 2, note that the only difference is the choice between the main staircase and the elevator. However, for Routing Variant 3, the algorithm recommends a very different route, where one should first traverse to another building (from ED to EC), going outdoors, then one should enter the EC building at the ground floor, go to the second floor, and follow the passage toward the ED building, to eventually access the destination point. The reason why the algorithm recommended this path is because the users specifically indicated that they wish to avoid crowded areas; when the other two routes became crowded, the algorithm recommended an alternative way, even if it is much longer.

The algorithm output is the sequence of nodes a user must follow to traverse the public space from one location to another. The number of times the algorithm offers that variant of response means how many times it gave that graph path as output. For Routing Variant 1, Figure 9 illustrates the first part of the response, as generated in the graphical interface. It shows the map of the entire ground floor of the ED building and the directions a person should follow to reach the destination, i.e., the way to get to the first floor of the ED building; from ED009, one must traverse hallway C, then hallway D, then take the stairs. The second part of the route is provided to the user as a different map, for the first floor. The maps for all the floors/areas that one should cross are given as a photo gallery in the interface, where one can scroll and view each part of the route.

### 5.2. Real-Life Loading Requirements

We hereby discuss the motivation for choosing the loads used for testing, such as to be as close to reality as possible. The tests refer to a particular kind of public space, a university campus. The graph used for these tests is already modeled after a real building. The question is how to estimate the number of newcomers in a university, to evaluate if the current architecture for our solution is good enough to serve them.

Real-life requirements for a university campus may result from studying the admission rates in each year from some well-known universities in the world, to deduce how many new students would need such routing services to orientate themselves in a university campus. University of Cambridge had 3528 newcomers in 2019, 3465 in 2018, and 3480 in 2017, according to [47]. University of Oxford admitted 3280 new students in 2019, 3309 in 2018, and 3270 in 2017 [48]. University College London had 9145 newcomers in 2019, 6110 in 2018, and 5885 in 2017. Eidgenössische Technische Hochschule Zürich had 3357 newcomers in 2020 [49]. Imperial College of London had 3045 freshmen in 2019, 2845 in 2018, and 2795 in 2017 [50]. University of Edinburgh received 7344 newcomers in 2019, 6346 in 2018, and 6221 in 2017 [51]. The number can be greater at the beginning of an academic semester or in the exams period, when the students in superior years may also need to find their way towards new laboratory or lecture rooms.

Then, let us also distribute the total number of students against the total possible number of years needed to obtain a bachelor’s degree. If a university also offers postgraduate studies, this data should be looked at as an outlier, because significantly less students choose to follow this study path; as noted in [52], in Austria as well as in Belgium, 1.0% of the students graduate from a postsecondary or postgraduate program each year, in Estonia or Czech Republic 0.7%, in Finland 1.1%, and in France 1.2%. Taking into consideration that the dropout rate of postgraduate studies is 12% in some cases [53] or 40% to 50% in others [54], one may conclude that the number of newcomers for postgraduate studies is negligible compared to the total number of students, who are mostly undergraduate. In this manner, one can extrapolate that from a university that has approximately 25,000 students, such as the one used for our tests, on a median span of 4 years for bachelor’s degree, 6000 new students come each year. 

Considering that not everyone accesses the routing services at the same time, the selection of loads used in our tests, i.e., the number of Virtual Users, follow the reality as closely as possible. For a different kind of public space, such as a transportation hub, these requirements may differ. 

## 6. Conclusions

The problem of pedestrian routing generally covers the following aspects: how to model the graph that corresponds to the real-world space, how to traverse the graph according to various cost functions, and how to consider other specific concerns for certain categories of people. It needs to map abstract mathematical knowledge to the concrete architectural elements, the circumstances of the moment, and the different pedestrians’ needs. This paper proposed a service-oriented design that considers all these aspects for delivering customized routing directions in a public space, by choosing from a set of policies. Two of them are driven by the community, by considering the votes of other pedestrians walking in the same public space, or by avoiding the places frequented by many other people. The other two are driven by data originating from multiple sources, such as sensors for measuring air quality or external weather monitoring services. In addition, a supplementary policy considers special needs for persons with reduced mobility. 

When adding supplementary policies, the tests for up to 5000 Virtual Users showed a small difference in response times (less than 1 s) and also in failure rates (less than 0.8%)—even if the execution was not carried out in a high-performance environment. Therefore, providing such software services, including the flexibility in choosing specific combinations of policies, is accessible when managing a public space. The loading tests proved that the routing services can support high loads of requests in a public space infrastructure modeled with a medium-sized granularity. 

Our recommendation when implementing this kind of algorithm is to start with the most suited graph model that represents the real public space. The representation granularity matters, and it is better to conduct an analysis beforehand, to determine what the specific points of interest are and to set the edges connecting the points of approximately the same size as in reality. Furthermore, it is recommended to determine the types of persons who use the public space. Might they have specific needs? What other information could be useful for them? This kind of analysis provides the best insights to create policies that correspond both to the reality and to its representation as a graph model. Special consideration should be given to how the weights are assigned, such as one policy does not cancel another. Nonetheless, the norming of the data values acquired from sensors is necessary for mapping them on the range of weights defined in the algorithms. This may be configured by the public space administrator, who may adjust them based on empirical knowledge. Lastly, our recommendations for assuring the right performance in very large public spaces are in the direction of an appropriate modularization strategy, deployment, and scaling, to offer even better performance and user experience under high loads.

## Figures and Tables

**Table 1:** Performance for 0-policy routing.

Case No.	VUs	Median Response Time (ms)	Avg. Response Time (ms)	Number of Requests	Requests per Second	Failure Rate (%)
1	1000	490	461.75	48,239	489.87	0.00
2	2000	1200	1568.56	100,326	468.17	2.44
3	3000	1300	2347.52	152,360	448.18	3.46
4	4000	1400	2846.88	216,316	440.03	3.27
5	5000	1400	3657.94	271,820	431.92	3.19

**Table 2:** Performance for 1-policy routing: Policy 2. Avoid crowded areas.

Case No.	VUs	Median Response Time (ms)	Avg. Response Time (ms)	Number of Requests	Requests per Second	Failure Rate (%)
1	1000	520	490.94	46,009	490.34	0.00
2	2000	1300	1738.12	99,027	435.18	2.71
3	3000	1400	2348.84	150,247	433.28	3.57
4	4000	1400	2976.30	210,319	445.93	3.39
5	5000	1400	3869.38	255,874	411.21	3.98

**Table 3:** Performance for 2-policy routing: Policy 2. Avoid crowded areas, plus Policy 3. Avoid polluted areas.

Case No.	VUs	Median Response Time (ms)	Avg. Response Time (ms)	Number of Requests	Requests per Second	Failure Rate (%)
1	1000	520 ms	582.80 ms	46,886	475.83/s	1.63%
2	2000	1300 ms	1770.20 ms	93,589	425.32/s	3.34%
3	3000	1400 ms	2434.81 ms	142,381	444.97/s	3.69%
4	4000	1500 ms	2987.47 ms	202,246	415.87/s	3.86%
5	5000	1500 ms	4549.83 ms	257,421	36,775.38/s	3.31%

**Table 4:** Responses for 100 requests with Policy 2.

Routing Variant	Routing Algorithm Response	Number of Times Recommended
1	“ed009”,“hallway_c_ed_1floor”,“hallway_d_ed_1floor”,“staircase_ed”,“hallway_d_ed_2floor”,“hallway_c_ed_2floor”,“ed114”	47
2	“ed009”,“hallway_c_ed_1floor”,“hallway_d_ed_1floor”,“elevator_ed”,“hallway_d_ed_2floor”,“hallway_c_ed_2floor”,“ed114”	47
3	“ed009”,“hallway_c_ed_1floor”,“hallway_d_ed_1floor”,“hallway_a_ed_1floor”,“entrance_ed_a”,“outside”,“entrance_ec”,“hallway_a_ec_1floor”,“staircase_ec_1”,“hallway_a_ec_2floor”,“entrance_ec_ed”,“hallway_a_ed_2floor”,“hallway_d_ed_2floor”,“hallway_c_ed_2floor”,“ed114”	4

## Data Availability

Not applicable.
